# Two years of COVID-19 vaccination in Nigeria: a review of the current situation of the pandemic: a literature review

**DOI:** 10.1097/MS9.0000000000001310

**Published:** 2023-09-13

**Authors:** Tolulope Joseph Ogunniyi, Basirat Oluwadamilola Rufai, Sunday Nguher Uketeh, Justice Kwadwo Turzin, Emmanuel Abiodun Oyinloye, Fortune Benjamin Effiong

**Affiliations:** a Department of Medical Laboratory Science, Kwara State University, Kwara State; b Faculty of Pharmacy, University of Ibadan, Oyo State; c Faculty of Pharmaceutical Sciences, University of Jos, Plateau State; d Department of Microbiology, Faculty of Science, Federal University, Oye-Ekiti, Ekiti State; e Directorate of Research, TORASIF, Calabar; f Department of Clinical Chemistry and Immunology, Faculty of Medical Laboratory Science, University of Calabar, Cross River State; g African Community for Systematic Reviews and Meta-analyses (ACSRM), Lagos, Nigeria; h Department of Biomedical Sciences, School of Allied Health Sciences, College of Health and Allied Sciences, University of Cape Coast, Ghana. PMB UCC, D-HUB, Cape Coast, Central Region, Ghana

**Keywords:** challenges, COVID-19 vaccination, effects, efforts, Nigeria

## Abstract

Curtailing COVID-19 outbreaks has been the major focus for many countries following the onset of the COVID-19 pandemic. Nigeria expanded its effort with the commencement of its vaccination program against COVID-19 in March 2021 after several less effective interventions as vaccine introduction was implemented. Following the introduction of the vaccines, Nigeria is expected to meet the worldwide COVID-19 eradication target of vaccinating 40% and 70% of the population, respectively, by the end of 2021 and 2022. Nigeria was unable to meet the target at the commencement of the program. The low vaccination rate, attributed to a low acceptance rate of vaccines, a lack of access to vaccines, poor communication, a weak cold-chain system, and inadequate infrastructure in the country, resulted in the complete vaccination of only 15% of the Nigerian populace as of 21 September 2022. To improve the vaccination rate, the COVID-19 Crisis Communication Centre was launched. Also, the implementation of delivery of service, logistics, accountability, supportive supervision, communication, and electronic management of immunization data scaled the vaccination rate to more than 54% of the target populace as of December 2022. Since the introduction of the COVID-19 vaccine, a substantial change in the prevalence and mortality rate has been perceived owing to the country’s progress toward achieving herd immunity against COVID-19. The country ascertained the percentage of cumulative deaths before the vaccination process to be 60.4%, which was reduced to 39.6% post-vaccination. In comparison, the percentage of confirmed COVID-19 cases was reduced from 58.3 to 41.7% post-vaccination. The authors recommend that the government and relevant public health authorities ensure meticulous documentation of the outcomes resulting from vaccination initiatives and facilitate the accessibility of this information to the general public to boost the vaccination rate.

## Introduction

Highlights

- In March 2021, Nigeria’s COVID-19 vaccination program commenced, with its objectives aligned to the goals of the global COVID-19 eradication program of vaccinating 40% and 70% of the population, respectively, by the end of 2021 and 2022.
- The major reasons for vaccine hesitancy were lack of self-assurance in the vaccine, fear of potential reaction, distrust of the government, limited knowledge about COVID-19, limited access to vaccines, weak cold-chain system and inadequate infrastructure.
- The Centers for Disease Control (CDC) in Nigeria commenced with two states as evidence of backing mass vaccination teams. Within three months, vaccination coverage increased by 15–25% in these two states.
- The messages of COVID-19 Crisis Communication Center (CRICC) focused on accessibility, safety, and efficacy which led to more over six million individuals receiving one shot of the vaccine, which improved demand for vaccinations and increased vaccination rates compared to the other campaign months.
- The Nigerian Government and its stakeholders implemented several innovative strategies, such as SCALES services to improve the accessibility of COVID-19 vaccine to citizens, hence a total of 111 985 403 vaccine doses were administered as at 7 March 2023 as reported by WHO.
- The percentage of cumulative deaths recorded before the vaccination process was 60.4%, reduced to 39.6% post-vaccination with zero deaths recorded from October 2022 until 7 March 2023.
- There is a need to intensify the awareness and campaign from the regional level to the community level in order to increase the citizens’ knowledge and awareness about COVID-19 vaccination.

The province of Wuhan announced the first COVID-19 case in December 2019, serving as the epicentre of the disease^1^. The WHO confirmed that COVID-19 has spread across the earth as a global pandemic on 11 March 2020, with 114 countries reporting 118 000 and 4291 cases and deaths, respectively^2^. The pandemic posed a massive impact on the socioeconomic status of individuals and disrupted the health systems and the world economy, which caused many countries to run into economic recession^3^. Nigeria recorded its first case in Lagos on 27 February 2020, when the global statistics just grew to over 5 million cases in more than 200 countries^4,5^. Nigeria, a nation boasting the highest population in Africa with over 214 million citizens, recorded a cumulative total of 162 593 confirmed cases of COVID-19 as of 28 March 2021^6,7^. In Nigeria, both Lagos State, the former capital, and the Federal Capital Territory registered the largest number of COVID-19 cases in the country^7^.

To curtail the pandemic, numerous interventions were established universally. Non-pharmaceutical interventions, which include travel bans, nose mask usage, regular handwashing, social distancing, and lockdowns^8^, helped ease the COVID-19 spread globally^9,10^. However, despite the non-pharmaceutical interventions, there is a necessity for pharmaceutical interventions like vaccines to achieve herd immunity against COVID-19^11^. Vaccination, the most effective form of public health intervention in curbing the problem of disease spread, helps prevent and eliminate contagious diseases and is also effective in reducing the mortality rate of disease^3^. In March 2021, Nigeria’s COVID-19 vaccination program commenced, with its objectives aligned with the goals of the global COVID-19 eradication program of vaccinating 40% and 70% of the population by the end of 2021 and 2022, respectively^12^. As of 21 September 2022, only 15% of the Nigerian populace had received a complete vaccination. The lower rate of vaccination is due to the challenges faced in the vaccination program, including inadequate cold-chain management, mistrust in the government, and communication breakdown in the course of the running of the program^13^. This paper aims to review the Nigerian COVID-19 vaccination program after 2 years of commencement, highlighting the challenges and efforts of COVID-19 vaccination as well as the effect of COVID-19 vaccination program on its prevalence.

## Challenges of vaccination in Nigeria

The COVID-19 vaccine development and subsequent vaccination of the population was the only effective strategy to control the pandemic^1^. The universal intervention to curb the pandemic was swift. In March 2021, Nigeria received its first set of AstraZeneca/Oxford vaccines (Covishield COVID-19 vaccines) mass-produced by the Serum Institute of India (SII) and distributed to different states. This was monitored using vaccine-tracking systems and immunization information systems to ensure proper allocation of the vaccines^14^. The low rate of COVID-19 vaccine acceptance observed at the initiation of the vaccination program in Nigeria was attributed to mistrust in the government and inadequate communication^15^. A study on vaccine hesitancy revealed that other major reasons for hesitancy were lack of self-assurance in the vaccine, fear of potential reactions, limited knowledge about COVID-19, limited access to vaccines, and the myth that Africans are automatically immune^16,17^. The majority of those who harbour this mistrust are rural residents who believe that the vaccine is unsafe and could harm them because the government produced it^16^.

Insufficient public relations efforts by the government to raise awareness about the accessibility of COVID-19 vaccinations during the initial phase of the vaccination program in Nigeria led to ineffective communication. This challenge hindered the understanding of the vaccine’s benefits among those who could not access information about the immunization rollout in their native languages. Consequently, safety concerns surrounding the vaccine emerged. In addition, Nigerians were required to schedule an online appointment before receiving a vaccination at the beginning of the program^18^. The vaccine gap in Nigeria was further exacerbated by limited internet access and a lack of technical knowledge among many Nigerians. The nation’s capability to vaccinate at least 40% of its populace by the end of 2021 was impacted by COVID-19^18^.

Furthermore, the effectiveness of the vaccine program was compromised due to a weak cold-chain system and inadequate infrastructure. Some COVID-19 vaccines require storage in ultra-cold temperatures to prolong their shelf life. To ensure the efficacy of Moderna and Pfizer vaccines during transportation and storage, they must be maintained at specific temperatures. Moderna vaccines should be kept between 2 and 8°C, while Pfizer vaccines require a temperature of −70°C before distribution. Due to the high prevalence of COVID-19 cases and a large population, the country’s available cold-chain storage space of 201 m^2^ was inadequate for a successful immunization campaign, as it fell short of the required 672 m^2^. Moreover, the lack of understanding among cold-chain personnel regarding the appropriate working system stood as a risk to the efficiency as well as the effectiveness of the cold-chain system. This, in turn, had a substantial effect on the overall achievement of the vaccination^18^.

## Efforts made to solve the challenges

To intensify the vaccination process, Nigeria began a nationwide mass vaccination campaign in November 2021, with the eligibility age set at 18 years and above and the estimated target population cumulating at around 112 million Nigerians, close to half of Nigeria’s population. To improve the vaccination program, the CDC gave its support to intensify the coverage of massive COVID-19 vaccination programs at the sub-national level^19^. The CDC in Nigeria commenced with two states as evidence of backing mass vaccination teams. Within 3 months, vaccination coverage increased by 15–25% in these two states^19^. Conveying provisional mass vaccination locations to hard-to-reach areas led to improvements in COVID-19 vaccination rates, Nigeria must at least triple this number in order to achieve fixed marks, as only about 200 000 doses are administered every day. The COVID-19 Crisis Communication Center (CRICC) was proven to be remarkable in a report submitted by the vaccine hesitancy study. The messages of CRICC focused on accessibility, safety, and efficacy^19^. The findings of the CDC’s social listening study helped establish risk messages clarifying the function of vaccines, which defend people from disease or mortality. As a result of these initiatives, in January 2022, more than six million individuals received one shot of the vaccine, which is an improvement demand for vaccinations and increased vaccination rates compared to the other campaign months. The Nigerian Government partners with the WHO, the Government of the United States, the United Nations Children’s Fund, other global and domestic partners, and non-governmental organizations to make sure vaccines, vaccination regularity, and other Prevention and Primary Health Care services get to all corners of the country to certify that everyone accepts basic health care^20^.

Furthermore, To advance the acceptance rate of COVID-19 vaccine, the Nigerian Government and its stakeholders implemented several innovative strategies, such as SCALES services (Service delivery, Communication, Accountability, Logistics, Electronic management of immunization data, and supportive supervision), to improve the accessibility of COVID-19 vaccine to citizens, with a strong focus on the susceptible populace and those who inhabit rural areas by incorporating additional health services alongside COVID-19 vaccination to fast-track the coverage of COVID-19 vaccination. The strategies implemented improve the vaccination program. A report stated by a health worker who is involved in outdoor vaccination outreach in Gwagwa village in Abuja revealed that individuals unwilling to receive the vaccine when the COVID-19 vaccination was introduced are currently accepting it. Also, at least ten individuals who had not accepted any dose of the vaccine received it every time they went out for the outreach. Individuals are also educated on the safety of receiving the vaccines to improve the rate of vaccination^21^. By December 2022, more than 54% (over 60 million individuals) of the target populace had been completely vaccinated^20^.

Furthermore, the WHO is still providing assistance to the government with regard to the technical management and coordination of COVID-19 vaccination efforts. This assistance includes tasks like managing the infodemic, generating evidence to support demand generation, offering supportive supervision, keeping an eye out for adverse events following immunization, and making available stipends and logistics for immunization teams across the states, among others. The government is also purchasing COVID-19 immunization supplies. The nation’s objective is to vaccinate at least 70% of the qualified populace with complete doses of vaccine by March 2023, coinciding with the worldwide target established by the WHO, in order to acquire herd immunity against COVID-19^21^.

## Effects of the vaccination on COVID-19 prevalence

Nigeria experienced an exponential elevation in the cases of COVID-19, and immediate intervention was needed to stop the pandemic. As of 28 March 2021, when Nigeria launched its vaccination campaign, there were 162 593 cumulatively established cases of COVID-19 reported in Nigeria^22^. This ranked Nigeria 5th among the utmost afflicted countries in Africa and 77th among the utmost afflicted countries in the world, while the cumulative deaths reported in Nigeria were 2048. In Nigeria, both Lagos State, the former capital, and the Federal Capital Territory registered the largest number of COVID-19 cases in the country^7^.

**Figure 1:**
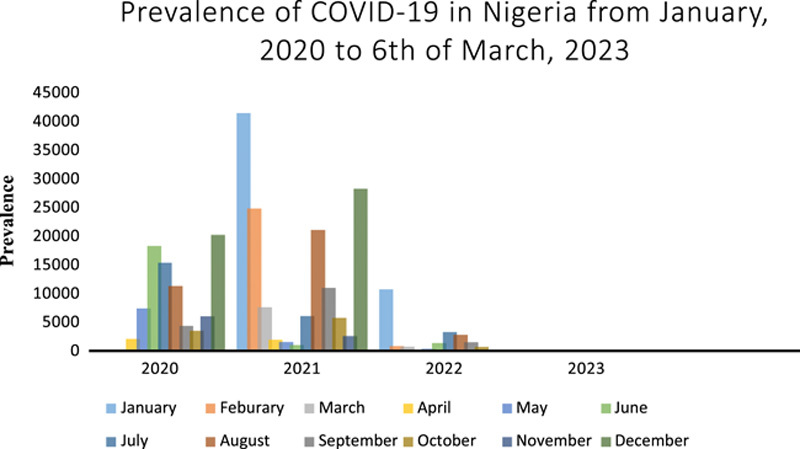
A chart denoting the prevalence since the inception of COVID-19 till 6 March 2023 in Nigeria according to WHO reports^9^.

The WHO report showed that as of 6 March 2023, 2 years after the start of the vaccination program, Nigeria had established an aggregate of 266 641 confirmed COVID-19 cases (Fig. 1). Additionally, as of 7 March 2023, the country has administered a total of 111 985 403 vaccine doses. This indicates a range of ~40–59 total doses administered per 100 people, with 20–39 individuals vaccinated with at least one dose per 100 people, 20–39 individuals vaccinated with the last dose of the primary series, and 10 individuals receiving a booster or additional dose per 100 people^23^.

**Figure 2:**
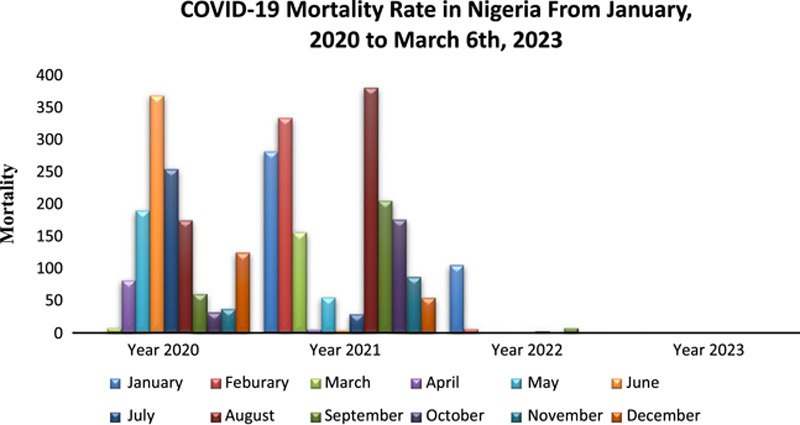
A chart denoting the mortality rate recorded since the inception of the COVID-19 pandemic till 6 March 2023, in Nigeria according to the WHO reports^9^.

The WHO reports also show that, as of 6 March 2023, Nigeria has recorded 3155 deaths since the inception of the pandemic. COVID-19 vaccination in Nigeria started in March 2021, and the report reveals a total of 1250 deaths were recorded from post-vaccination till 6 March 2023(Fig. 2). This indicates that the vaccination process had a positive impact on curbing the pandemic in Nigeria through the reduction in the prevalence and the number of deaths associated with the disease since the inception of the vaccination. Zero deaths have been recorded from October 2022 until 6 March 2023. The percentage of cumulative deaths recorded before the vaccination process was 60.4%, reduced to 39.6% post-vaccination, while the percentage of confirmed COVID-19 cases reduced from 58.3 to 41.7% post-vaccination^23^.

## Recommendation

To further strengthen the vaccination against COVID-19 in Nigeria, there is a need to improve the citizens’ trust by ensuring effective communication. There is a need to intensify the awareness and campaign from the regional level to the community level in order to increase the citizens’ knowledge and awareness about COVID-19 vaccination. Vaccine information should be effectively translated into local languages and disseminated through various media channels such as radio, television, newspapers, and informative posters to ensure a comprehensive reach. This approach will cater to individuals who might not possess advanced technical knowledge, granting them the necessary access to vital vaccine-related information. The vaccination program should be brought closer to the community, particularly in hard-to-reach communities. This can be achieved through the use of community health centres that are situated within the community. More support for the vaccine storage systems through the collaboration of government and non-governmental organizations will be essential to reaching the goals of COVID-19 and future vaccination programs. The government and relevant public health authorities should ensure meticulous documentation of the outcomes resulting from vaccination initiatives and facilitate the accessibility of this information to the general public to boost the vaccination rate. This paper has the limitation of not addressing the long-term effects of COVID-19 vaccines on the dynamics of the pandemic and the effects of vaccination on post-COVID-19 syndrome in Nigeria due to insufficient data; these can serve as the basis for subsequent research.

## Conclusion

Ever since the outbreak of COVID-19, several interventions have been rolled out to curtail the pandemic. Yet the COVID-19 vaccine was needed to reduce its spread drastically and achieve herd immunity. Nigeria commenced its vaccination against COVID-19 in March 2021, with the targets of inoculating 40% and 70% of the population by the end of 2021 and 2022, respectively. However, Nigeria faced challenges throughout this period in achieving these global vaccination program targets. These setbacks were primarily attributed to a low vaccine acceptance rate, limited access to the vaccine, ineffective communication, weak cold-chain systems, and inadequate infrastructure.

Several measures were implemented within the 2 years following the commencement of the COVID-19 vaccination to address these challenges. These included mass vaccination campaigns, establishing the COVID-19 Crisis Communication Centre (CRICC) and enhancing service delivery, accountability, logistics, communication, supportive supervision, and electronic management of immunization data. These efforts resulted in over 54% of the population being vaccinated as of December 2022. We conclude that the impact of the vaccination process has been significant, leading to zero recorded cases of COVID-19 in 2023.

## Ethical approval

None.

## Consent

None.

## Sources of funding

None.

## Author contribution

T.J.O.: led the research work, writing- original draft, final approval and agreeing to the accuracy of the work. B.O.R.: writing—original draft, final approval, compilation of the paper and agreeing to the accuracy of the work. S.N.U.: writing—original draft, final approval and agreeing to the accuracy of the work. J.K.T.: writing—original draft, final approval and agreeing to the accuracy of the work. E.A.O.: conceived the idea, reviewed the work and agreeing to the accuracy of the work. F.B.E.: reviewed the work, final approval, final proofreading of the paper and agreeing to the accuracy of the work.

## Conflicts of interest disclosure

None.

## Research registration unique identifying number (UIN)

1. Name of the registry: Not Applicable.
2. Unique Identifying number or registration ID: Not Applicable.
3. Hyperlink to your specific registration (must be publicly accessible and will be checked): Not Applicable.

## Guarantor

Tolulope Joseph Ogunniyi, Basirat Oluwadamilola Rufai, Sunday Nguher Uketeh, Justice Kwadwo Turzin, Emmanuel Abiodun Oyinloye, Fortune Benjamin Effiong.

## Data availability statement

Publicly available.

## Provenance and peer review

Not commissioned, externally peer-reviewed.
